# Liquefaction of Peanut Shells with Cation Exchange Resin and Sulfuric Acid as Dual Catalyst for the Subsequent Synthesis of Rigid Polyurethane Foam

**DOI:** 10.3390/polym11060993

**Published:** 2019-06-04

**Authors:** Qinqin Zhang, Weisheng Chen, Guojuan Qu, Xiaoqi Lin, Dezhi Han, Xiaofei Yan, Heng Zhang

**Affiliations:** 1 Shandong Provincial Key Laboratory of Biochemical Engineering, College of Marine Science and Biological Engineering, Qingdao University of Science and Technology, Qingdao 266042, China; qqzhang@qust.edu.cn (Q.Z.); weisheng_chen@163.com (W.C.); Y30181018@mail.ecust.edu.cn (G.Q.); lxq15621496181@163.com (X.L.); hgzhang@sina.com (H.Z.); 2 College of Chemical Engineering, Qingdao University of Science and Technology, Qingdao 266042, China; 3 Qingdao Institute of Bioenergy and Bioprocess Technology, Chinese Academy of Sciences, Qingdao 266101, China; yanxf@qibebt.ac.cn

**Keywords:** lignocellulosic biomass, peanut shells, liquefaction reaction, strong-acid cation exchange resin, polyols, rigid polyurethane foam

## Abstract

The conversion of lignocellulosic biomass from renewable raw materials to high value-added fine chemicals expanded their application in biodegradable polymers materials synthesis, such as polyurethanes and phenolic resin, etc. In this work, the strong-acid cation exchange resin and sulfuric acid as the dual catalyst offered an effective way to catalyze the liquefaction reaction of the peanut shells. The properties of liquefied products were characterized by means of hydroxyl value, viscosity and solubility tests, while the properties of peanut shells and liquefaction residue were analyzed by means of ATR-FTIR, TG and SEM techniques. The results indicated that the liquefied products could be completely dissolved in deionized water, methanol and polyethylene glycol, respectively, and they could be a preferable substitution of petrochemical polyols as soft segments to synthesize the rigid polyurethane foams. Moreover, the cellulose and hemicellulose in the peanut shells were easily decomposed into smaller molecules via the breakage of the C–O bond besides five-membered and hexatomic ring, while the lignin could be degraded via the breakage of the C–O chemical bonds of β-O-4, 4-O-5 and dibenzodioxocin units. The fabricated rigid polyurethane (RPU) foam, containing higher percentage of open pores with uniform size, can be potentially utilized for flower mud and sound-absorbing materials.

## 1. Introduction

Polyurethanes (PUs), as an extensive variety of polymers, are widely applied in foams, elastomers, coatings, adhesives and sealants. Polyurethane foams are the most important commercial products and generally divided into flexible, semi-flexible and rigid foams, due to the configuration of the foam cell, rigidity and density. Typically, rigid polyurethane (RPU) foams widely serve in the heat and cold preservation field including household refrigerators, industrial pipelines and building layers, depending on their particular incorporation of preferable thermal isolation and mechanical properties [1].

The synthesis of PUs is determined through the reaction of isocyanates with polyols to from urethane (–NH–(C=O)–O–) linkages. Nowadays, the isocyanates and polyols are mainly prepared and supplied from petroleum-derived feedstocks [2]. According to the total amount of global polymer consumption in 2011, 5% arises from polyurethane raw materials—isocyanates and polyols [3]. Due to the fast consumption of petroleum feedstocks, it is urgent to explore alternative biomass resources which can be further translated into basic chemical raw materials. As renewable and globally available resources, biomass possesses ignorable sulfur and other pernicious elements, and can be identified as a carbon-neutral resource without the net increment of the CO_2_ concentration in the atmosphere [4].

The lignocellulosic biomass as the abundant raw material on the earth, consists of polysaccharide polymers (cellulose, hemicellulose) and an aromatic polymer (lignin). Their molecules, with plenty of phenolic and ester hydroxyl groups, could react with the isocyanate functional group to generate polymers with urethane bonds. Typical lignocellulosic biomass, such as cellulose derivatives [5], lignin derivatives [6], straws [7,8,9], barks [10], bagasse [11], wood [12,13,14,15,16] and bamboo [17] has been liquefied by appropriate solvents (various glycols) to produce bio-based polyols, which was used as raw materials for preparing PUs. In general, the conventional liquefaction reaction of lignocellulosic biomass is catalyzed by acid, especially sulfuric acid [8,13,15,18]. The catalytic efficiency of sulfuric acid is usually desirable; however, the influence of excessive sulfate ion on the qualities of subsequent polyurethanes is still undetermined. 

Compared to the liquid acid, the strong-acid cation exchange resin (SCER), which is a renewable catalyst in industrial application, can be easily recovered and reused. It also exhibited premium catalytic performance in the liquefaction process of lignocellulosic biomass. As reported in previous work, researchers had investigated the gel-type SCER for separating small molecule compounds, such as monosaccharides and organic acids, from lignocellulosic hydrolysates [19,20]. However, to the best of our knowledge, less research on the investigation of SCER for the liquefaction of lignocellulosic biomass has been reported.

The main objective of this study was to (1) liquefy the peanut shell powder with SCER and sulfuric acid as the dual catalyst and investigate the influences of processing parameters to optimize liquefaction conditions, (2) analyze the properties of liquefied products and comparatively evaluate the properties of peanut shells and liquefaction residue, and (3) synthesize and characterize the RPU foams from liquefied products. The present work would offer an alternative way to liquefy lignocellulosic biomass for the subsequent synthesis of bio-based RPU foams.

## 2. Materials and Methods

### 2.1. Materials 

Polymeric methylene-4,4′-diphenyl diisocyanate (PMDI) was purchased from Wanhua Chemical Group Co., Ltd, China. Polyethylene glycol 400 (PEG 400), glycerol, sulfuric acid (98.3 wt. %), tetrahydrofuran, ethanol, cyclohexane, methanol, polyethylene glycol 2000 and n-Hexane with CP or AR grade were obtained from Sinpharm Chemical Reagent Co., Ltd., Shanghai, China. Triethylene diamine, stannous octoate and silicone-based surfactant were supplied by Air Products & Chemicals, Inc. (Allentown, PA, USA). The above reagents were employed as supplied without further purification. 

The peanut shells were collected from the cropland in Rizhao city, Shandong province, China. Acid insoluble lignin (GB/T 2677.8-94) and ash contents (GB/T 2677.3-93) in peanut shells was 30.9% and 3.1%, respectively. The peanut shells possessed an elemental composition (characterized by vario EL III element analyzer, Elementar Analysensysteme GmbH, Langenselbold, Germany) corresponding to C (47.20%), H (5.6%) and N (0.4%), respectively. After careful washing and drying, the peanut shells were milled in a high speed grinder with rotation speed of 10,000 rpm and the obtained powder with particle dimensions under 60 mesh was collected for the following liquefaction. The wet-type SCER (Rohm and Haas Company, Philadelphia, PA, USA) was dried at 105 °C overnight for total water removal before use, of which properties are listed in Table 1.

### 2.2. Liquefaction of Peanut Shells 

The liquefaction experiment was conducted in a 100 mL three-necked flask fitted with a mechanical stirrer, thermometer and condenser. Firstly, peanut shells, glycerol and PEG 400 were poured into the flask, which was located in an oil bath with a temperature of 120–160 °C. Then, the sulfuric acid as strong acid catalyst was subsequently added into the above mixture with constant stirring for 0.5 h, followed by the addition of SCER as solid acid catalyst with constant stirring for another 1.0 h. Finally, the liquefied mixture was poured into a Buchner funnel under vacuum (0.09 MPa). The liquefaction residue was obtained after being completely washed by deionized water and dried under 110 °C overnight. The liquefied products were obtained after the complete evaporation of water. The liquefaction yield was calculated on the basis of the following equation:

(1) $$\mathrm{Liquefaction}\;\mathrm{yield}=(1-\frac{m_{c}-m_{p}-m_{r}}{m_{o}})\times 100\%$$

where *m_c_* represents the total constant weight of the liquefaction residue and the filter paper, while *m_p_*, *m_r_*, *m_o_* represent the constant weight of the filter paper, dried SCER and original peanut shells, respectively.

### 2.3. RPU Foam Preparation

The RPU foam was synthesized in a 500 mL plastic beaker via the one-step synthesis [21]. The RPU foam formulation in this work was as follows: Liquefied products of 100 wt. %, physical blowing agent (water at 2.5 wt. %), stannous octoate (0.3 wt. %), triethylene diamine (1.0 wt. %) and silicone-based surfactant (2.5 wt.%). The amounts of the above-mentioned materials were determined relative to the total weight of liquefied products. Moreover, the PMDI was used with an equivalent ratio (isocyanate group to hydroxyl group) of 1/1. The synthesis procedure of RPU foam was as follows: Firstly, liquefied products, blowing agent, catalysts and surfactant were weighed, then transferred into the plastic beaker and mixed by stirring with the rate of 800 rpm for 28–32 s to get the homogeneous mixture. Then, the PMDI was introduced to above mixture and stirred vigorously for another 90–120 s. Finally, the beaker was located on a flat and the foam was allowed to cure under room temperature (25 ± 2 °C) for at least 72 h.

### 2.4. Characterization

The hydroxyl value measurement of liquefied products was conducted using a PHS-3C acidometer (Shanghai INESA Scientific Instrument Co., Ltd, Shanghai, China) according to specification of GB/T 12008.3-2009 with an electric potential titrimetric method. The viscosity of liquefied products was implemented using a rotational viscometer (Brookfield Programmable DVIII Viscometer) under a rotation rate of 30 rpm. The solubility of liquefied products was measured in common organic and inorganic solvents. Liquefied products of 1mL was injected into each solvent of 20 mL to form the mixture, which then was filtrated over the filter paper. Thermogravimetry (TG) data was recorded from Netzsch Simultaneous Thermal Analyzer STA 449F5 Jupiter with the heating speed of 10 °C min^−1^ in the temperature range of 30–800 °C for studying the thermal degradation of peanut shells and liquefaction residue. Attenuated total reflectance Fourier transform infrared (ATR-FTIR) spectra of peanut shells and liquefaction residue were collected from a spectrometer of Bruker VERTEX 70 (Bruker Optik GmbH, Ettlingen, Germany) in the range from 4000 to 600 cm^−1^ with 32 scans and a resolution of 2 cm^−1^. The morphologies of peanut shells, liquefaction residue and RPU foam were examined on a scanning electron microscope (SEM, Hitachi S-4800, Hitachi High-Technologies Corp., Tokyo, Japan) with a backscattered electron detector running at 3 kV. The compressive strength test of RPU foam was conducted according to GBT 8813-2008 on an electronic universal testing machine H10KS (Hounsfield Test Equipment Ltd., Redhill, Surrey, UK) with a loading speed of 5 mm min^−1^.

## 3. Results and Discussion

### 3.1. Optimization of Liquefaction Conditions 

The lignocellulosic biomass degradation in organic solvents primarily refers to solvolysis [11]. The solvolysis rate could be promoted with the aid of strong acids as catalyst, which could elevate the biomass breakup at early stage and prevent intermediates repolymerization at a later stage [22,23]. Hence, in this study, the concentrated sulfuric acid and SCER were employed as a dual catalyst to enhance the liquefaction process of peanut shells. It can be seen from Table 1 that the average pore diameter of the SCER is only 19 nm. Therefore, when the SCER was solely utilized as the catalyst during the liquefaction process, the pores of SCER could be blocked by peanut shell powder, resulting in a low liquefaction yield. However, the problem can be resolved by the combination of sulfuric acid and SCER as a dual catalyst. The sulfuric acid was firstly added in the liquefaction system for catalyzing the peanut shells breakup to form primary products, followed by the addition of SCER to further accelerate the degradation. Furthermore, the SCER can be easily recovered and reused, indicating that this could be feasible and a potential method for practical application on the effective liquefaction of lignocellulosic biomass.

**Figure 1:**
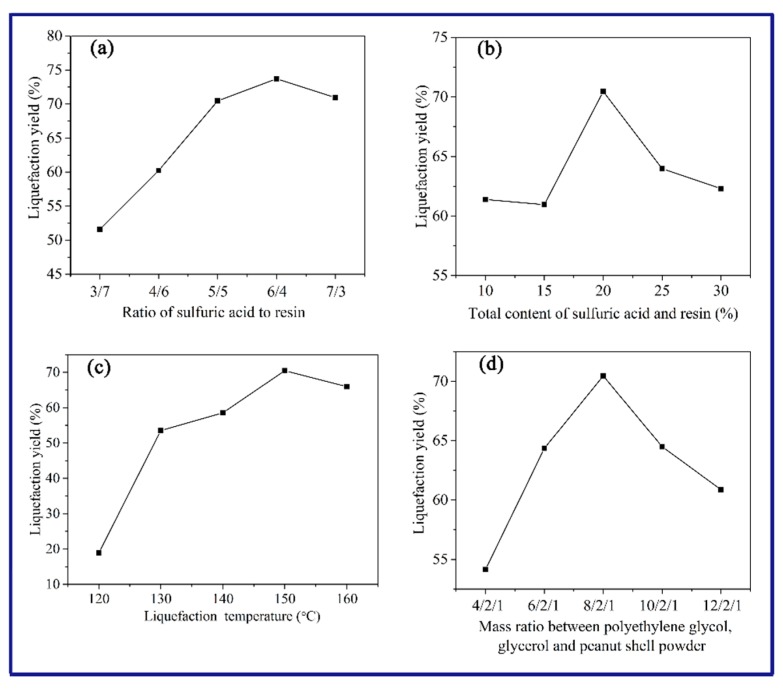
Liquefaction yield under different reaction parameters of (**a**) ratio of sulfuric acid to resin, (**b**) total catalysts content, (**c**) liquefaction temperature and (**d**) PEG-G-PSP (polyethylene glycol–glycerol–peanut shell powder ratio.

Figure 1 shows the liquefaction yield with respect to the ratio of sulfuric acid to resin, total catalysts content, liquefaction temperature and mass ratio of polyethylene glycol, glycerol and peanut shell powder (defined as PEG-G-PSP ratio). It is found from Figure 1a that the liquefaction yield increases with the increase in the ratio of sulfuric acid to resin, and then nearly reaches a plateau. When the ratio is <5/5, the sulfuric acid with low content could not effectively catalyze the degradation of the peanut shells to form primary products in the earlier stage, leading to the low liquefaction efficiency in the later stage. However, liquefied products with high content of sulfuric acid may dramatically affect the performance of as-synthesized polyurethane [21]. Besides, the optimum total catalysts content is 20% (relative to mass of peanut shells powder) in Figure 1b. This indicates that the dual catalyst system exhibited high catalytic dehydration activity at low content in degradation process. The investigation of liquefaction temperature in Figure 1c illustrates that the liquefaction yield increased with increase in temperature and reached maximum value at the temperature of 150 °C. As the temperature further rises to 160 °C and beyond, the liquefied products may be transformed into residue by cyclization and aggregation reaction, leading to the decrease in liquefaction yield. Moreover, it can be seen from Figure 1d that the PEG-G-PSP ratio has a dramatic influence on the liquefaction yield, and the liquefaction yield has a maximum value of 70.6 % when the PEG-G-PSP ratio is 8/2/1. Hence, the liquefaction reaction was performed under the optimum liquefaction conditions in the following experiments.

### 3.2. Properties of Liquefied Products 

#### 3.2.1. Hydroxyl Value and Viscosity Property

In general, the appropriate hydroxyl value for RPU foam synthesis is in the range of 200–550 mgKOH g^−1^ [24]. The hydroxyl value of liquefied products and liquefaction solvents (mass ratio of PEG 400 to glycerol = 8/2) are 472.05 mgKOH g^−1^ and 337.06 mgKOH g^−1^, respectively. Thus, this implies that the liquefied products contain large amounts of aliphatic and aromatic hydroxyl after the liquefaction of peanut shells, and could be used to substitute petrochemical-based polyether polyols such as PEG 400 for the synthesis of RPU foams. Moreover, the viscosity of liquefied products is 46 mPa s at 25 °C, which is close to that of liquefaction solvents (41 mPa s), indicating that liquefied products are feasible as soft segments for synthesis of RPU foams. The viscosity of liquefied products could be tuned by changing the type of liquefaction solvents. 

#### 3.2.2. Solubility Tests

**Figure 2:**
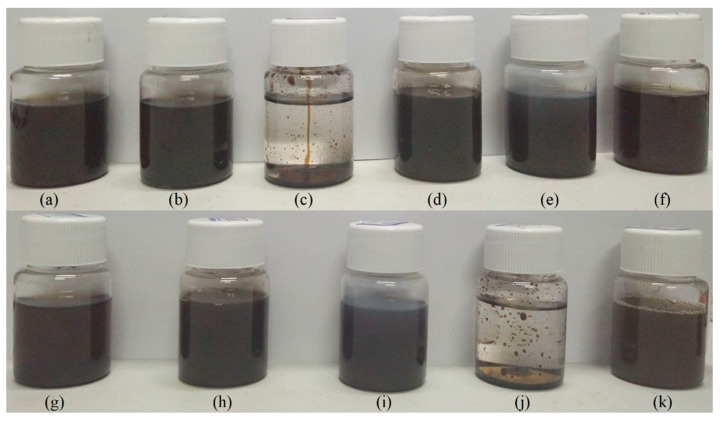
The solubility of liquefied products in common solvents: (**a**) tetrahydrofuran, (**b**) ethanol, (**c**) cyclohexane, (**d**) methanol, (**e**) deionized water, (**f**) glycerol, (**g**) glycerol/ deionized water (*v*/*v* = 1/1), (**h**) PEG 2000, (**i**) PEG 400, (**j**) n-Hexane, (**k**) methanol/deionized water (*v*/*v* = 1/1).

The molecular of peanut shells contains phenolic oxhydryl groups and carboxyl groups, leading to the excellent polarity and reaction activity. Thus, as shown in Table 2 and Figure 2, the liquefied products were insoluble in nonpolar solvent, such as n-hexane and cyclohexane, while they were entirely soluble in deionized water, methanol, PEG 400 and PEG 2000. These results, along with the hydroxyl value, offer the strong evidence that the liquefied products as the soft segments are suitable for preparing polyurethane materials.

### 3.3. Properties of the Peanut Shells and Liquefaction Residue

#### 3.3.1. Thermal Behaviour 

**Figure 3:**
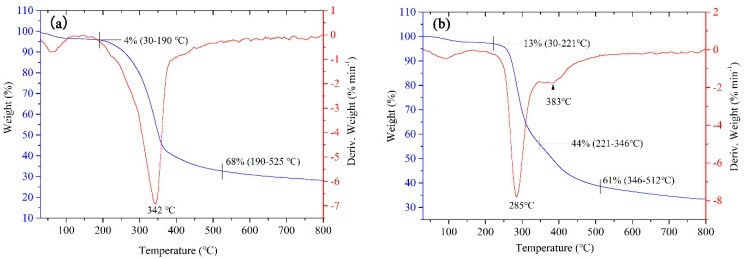
TG and DTG curves of (**a**) peanut shell powder and (**b**) liquefaction residue.

The thermal stability of the peanut shells and liquefaction residue was evaluated by TG and differential thermogravimetry (DTG) characterization (Figure 3). The small fluctuation in the earlier stage could be attributed to the water evaporation in the samples. The pyrolysis of peanut shells and liquefaction residue starts from 190 °C and 221 °C, where the mass loss is 4% and 13%, respectively. As shown in Figure 3a, the TG curve of peanut shells shows the significant weight loss in the temperature range of 190 °C and 525 °C with the obvious DTG peak at 342 °C, which could be primarily attributed to the degradation of the hemicellulose (around 250 °C), cellulose (around 300 °C) and an extensive pyrolysis of lignin (around from 200 °C to 500 °C) [25,26,27,28]. Furthermore, as shown in Figure 3b, the maximum weight loss peak in DTG curve of liquefaction residue locates at the temperature of 285 °C, which is much lower than that (342 °C) of the peanut shells. This implies that the dual catalyst system is effective for the partial degradation of hemicellulose and cellulose and deeper degradation of lignin in the peanut shells into polyols in comparison with the single sulfuric acid, leading to the easy pyrolysis of the undecomposed constituents in the liquefaction residue during the TG test. As the temperature is higher than 525 °C for peanut shells and 512 °C for liquefaction residue, the decomposition process becomes gentle, which is in accordance with a previous report [29]. The final char content for peanut shells and liquefaction residue is 28.16 % and 33.35%, respectively, indicating that the content of the stable components in liquefaction residue is higher than in peanut shells.

#### 3.3.2. Functional Groups of the Peanut Shells and Liquefaction Residue

**Figure 4:**
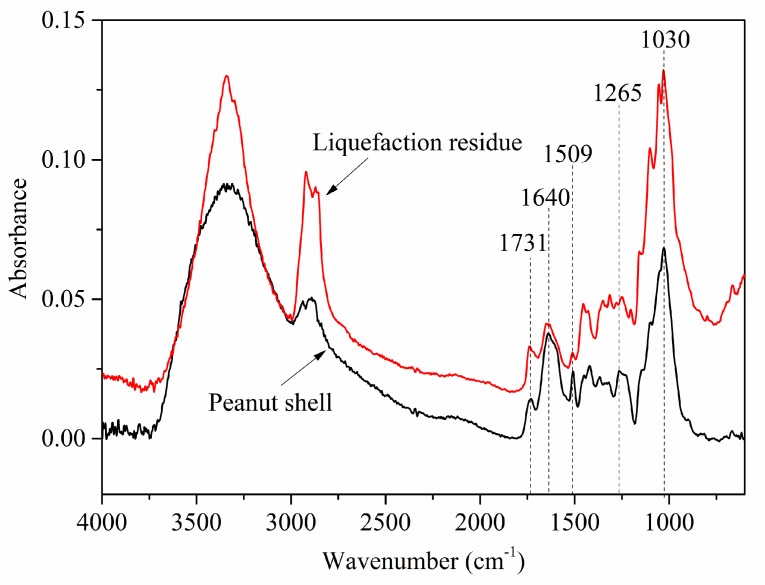
ATR-FTIR spectra of peanut shell powder and liquefaction residue.

Figure 4 shows the ATR-FTIR spectra of peanut shells and liquefaction residue. ATR-FTIR spectra for the lignocellulosic biomass are invariably complicated, because of multifarious functional groups involved in the molecules of cellulose, hemicellulose and lignin. It can be seen that most bands of peanut shells and residue locate in the similar position. The extensive absorption bands at 3600–3100 cm^−1^ are attributed to OH stretching vibration of phenolic hydroxyl group from lignin or alcoholic hydroxyl group from cellulose and hemicellulose [30,31]. The bands around 2860–2970 cm^−1^ are ascribed to the C–H stretching vibration from methyl and methylene groups (alkyl and aromatic) [32].

**Figure 5:**
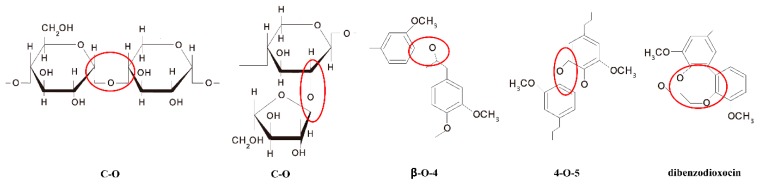
C–O bonds in polysaccharide and β-O-4, 4-O-5, and dibenzodioxocin bonds in lignin.

The middle absorption bands at around 1731 cm^−1^ can be found for peanut shells and liquefaction residue. It is mostly assigned to ester carbonyl group in xylan [33,34]. Above peak intensity of liquefaction residue remains unchanged, which indicates liquefaction residue still contains some part of undecomposed cellulose, hemicellulose and lignin. Although the catalytic effect of sulfuric acid and SCER has promoted the solvolysis of polyethylene glycol and glycerol on the ester linkage in the xylan, the liquefaction residue still contains undegradable constituents with bigger molecules, which are more difficult to decompose into smaller molecules. Absorption bands at around 1640 cm^−1^ and 1509 cm^−1^ arise from the C=C stretching vibration of the aromatic skeletal in lignin [33,35]. Apparently, those two peaks of liquefaction residue become weaker in comparison with that of peanut shells, suggesting that the lignin was partly degraded in the liquefaction process, which is consistent with the TG analysis. The absorption peak at 1264 cm^−1^ responds to the C–O–C stretching vibration in β-glucosidic bonds of cellulose and hemicellulose and guaiacyl alcohol units in lignin. Compared to the moderate absorption intensity of peanut shells, it shows a very weak absorption intensity and a shift in position to 1273 cm^−1^ for liquefaction residue. This illustrates that the cellulose and hemicellulose was decomposed into smaller molecules via the breakage of C–O linkage, while the lignin was degraded via the breakup of the dominant bonds of β-O-4, 4-O-5, and dibenzodioxocin units (as illustrated in Figure 5), which is in accordance with the previous reports [36,37]. An overlap peaks for strong absorption at around 1030 cm^−1^ are attributed to the C–H deformation vibration of the aromatic ring. The absorption for liquefaction residue becomes stronger than that of peanut shells, which can be observed from the relative intensity of OH stretching at 3600–3100 cm^−1^. It is found that the aromatic ring content rises in the liquefaction residue with comparison to the peanut shells after typical bonds cleavage, due to no breakage of C=C in aromatic skeleton [38,39,40]. 

#### 3.3.3. Morphology of the Peanut Shells and Liquefaction Residue

**Figure 6:**
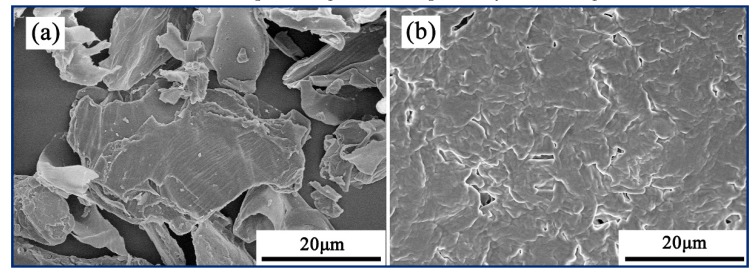
SEM images of (**a**) peanut shells and (**b**) liquefaction residue.

The SEM images of the peanut shells and liquefaction residue are shown in Figure 6. The regular fiber bundles for the peanut shell sample can be visibly observed. However, the liquefaction residue exhibits the rough surface and contains numerous fragments of cell wall components after the liquefaction process. This indicates that the fiber bundles in the peanut shells are broken during the liquefaction process and the undecomposed components in liquefaction residue lost their original ordered structure while the decomposed segments are split away off the original fibers.

### 3.4. RPU Foam from Liquefied Products

**Figure 7:**
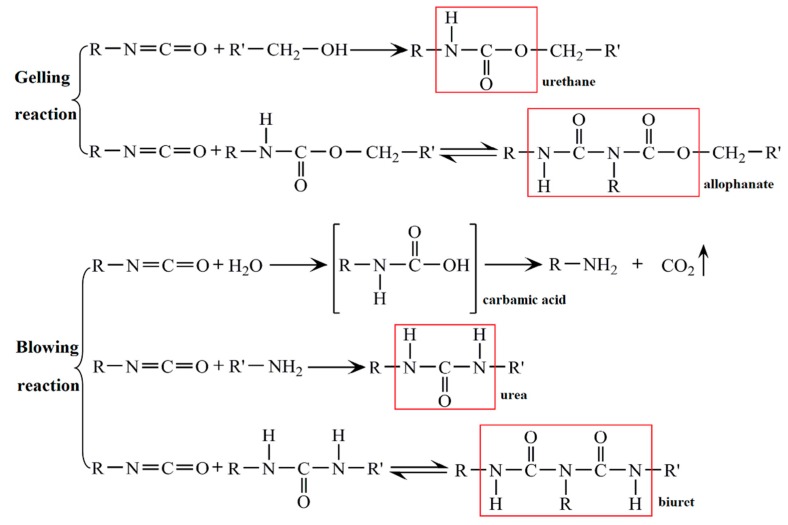
Scheme of gelling and blowing reactions in the synthesis of polyurethane foam.

**Figure 8:**
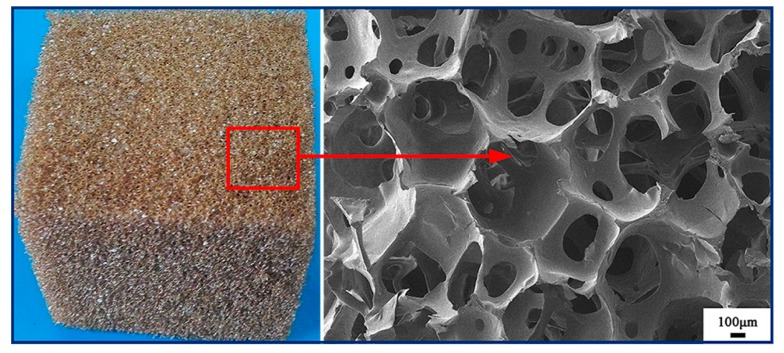
Camera photo and SEM image of rigid polyurethane (RPU) foams from liquefied products.

As shown in Figure 7, polyurethane can be successfully synthesized via the reaction of liquefaction products (–OH) and isocyanates (–NCO) by forming urethane linkages [–NH–(C=O)–O–]. Except for urethane linkages, allophanate linkages are probably produced because of the reaction between excess diisocyanates and N–H bond in urethane groups. Furthermore, the dimerization and trimerization reactions of diisocyanates can also take place [41]. The microstructure of foams is related to the above complicated reaction process. The camera photo and SEM image of the RPU foam synthesized from the liquefied products are shown in Figure 8. The presence of open cells can be clearly observed. In the bubbles formation process, the gelling reaction rate is slow because of the lower reaction activity of liquefied products in comparison with the petrochemical polyols. Therefore, the bubbles are easily escaped from the matrix before it forms the firm struts. Finally, the gelation and blowing reaction reach an equilibrium, resulting in the formation of RPU foam with uniform pore size. Furthermore, RPU foam sample displays a three-dimensionally ordered structure with well-ordered air spheres and interconnected walls. From an application consideration, the RPU foam with higher percentage of open pores is potentially utilized for flower mud and sound-absorbing materials. 

**Figure 9:**
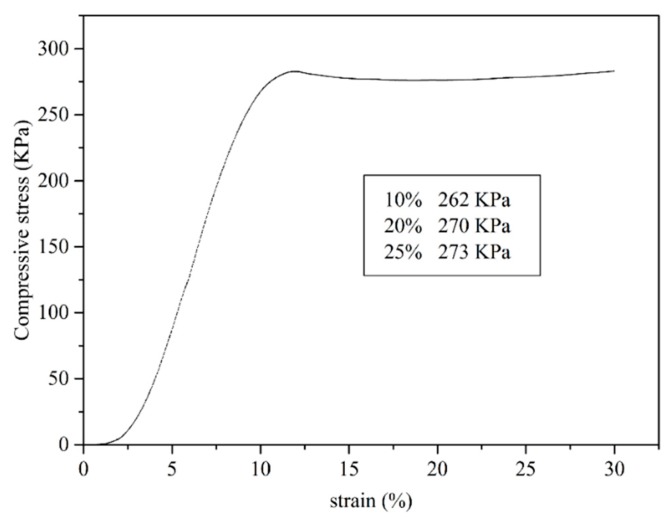
Stress-strain curve of the prepared RPU foam.

The mechanical property, especially compressive strength, is more relevant with application performance. It can be seen from Figure 9 that the compressive strength of RPU foam is around 270 KPa at ambient temperature when the strain is higher than 10%, which is higher than that (190 KPa) of the RPU foam prepared from the liquefied products if only the sulfuric acid was used as the single catalyst presented in our previous work [21]. Generally, compressive strength requirement for the flower mud or sound-absorbing materials is lower than that for application in building insulation (80 kPa to180 kPa), which is classified by China Standard of GBT 21558-2008. Therefore, the RPU foam fabricated in this study could possess the superior performance in the application of flower mud or sound-absorbing materials.

## 4. Conclusions

The SCER and sulfuric acid as a dual catalyst exhibited an effective way for liquefaction of the peanut shells. The optimum liquefaction conditions were determined with ratio of sulfuric acid to resin of 5/5, total catalyst content of 20%, liquefaction temperature of 150 °C and PEG-G-PSP ratio of 8/2/1. The liquefied products had sufficient solubility in deionized water, methanol, polyethylene glycol 400. Furthermore, the cellulose and hemicellulose in the peanut shells were easily decomposed into smaller molecules via the breakage of the C–O linkage, while the lignin was degraded via the breakage of the dominant bonds of β-O-4, 4-O-5, and dibenzodioxocin units. The SEM images revealed that the fiber orientation of peanut shells was dramatically changed during the liquefaction process and the undecomposed constituents in liquefaction residue lost their original regular structure while the decomposed segments were split away from the original fibers. The RPU foam prepared from liquefied products possessed higher percentage of open pores with uniform size, which could be potentially utilized for flower mud and sound-absorbing materials.

## Figures and Tables

**Table 1:** Properties of cation exchange resin.

Items	Properties and Operating Conditions
Physical appearance	Dark brown, spherical particle
Ionic form as shipped	Hydrogen (≥98%)
Concentration of acid sites	≥2.98 eq kg^−1^
Moisture holding capacity	51.0%–55.0% (H^+^ form)
Particle size	0.58–0.75 mm
Fines content	<0.355 mm, 2.0% max
Surface area	49 m^2^ g^−1^
Average pore diameter	19 nm
Maximum operating temperature	170 °C

**Table 2:** Solubility of liquefied products in conventional solvents.

Solvents	Solubility
n-Hexane	Insoluble
Cyclohexane	Insoluble
Ethanol	Partly soluble
Glycerol	Partly soluble
Glycerol/ deionized water(*v*/*v* = 1/1)	Partly soluble
Tetrahydrofuran	Mostly soluble
Methanol/deionized water (*v*/*v* = 1/1)	Fully soluble
Deionized water	Fully soluble
Methanol	Fully soluble
Polyethylene glycol 400	Fully soluble
Polyethylene glycol 2000	Fully soluble
